# *In situ* production of active vitamin B12 in cereal matrices using *Propionibacterium freudenreichii*

**DOI:** 10.1002/fsn3.528

**Published:** 2017-11-12

**Authors:** Bhawani Chamlagain, Tessa A. Sugito, Paulina Deptula, Minnamari Edelmann, Susanna Kariluoto, Pekka Varmanen, Vieno Piironen

**Affiliations:** ^1^ Department of Food and Environmental Sciences University of Helsinki Helsinki Finland

**Keywords:** barley malt and flour, fermentation, *Propionibacterium freudenreichii*, vitamin B12, wheat aleurone

## Abstract

The *in situ* production of active vitamin B12 was investigated in aqueous cereal‐based matrices with three strains of food‐grade *Propionibacterium freudenreichii*. Matrices prepared from malted barley flour (33% w/v; BM), barley flour (6%; BF), and wheat aleurone (15%; AM) were fermented. The effect of cobalt and the lower ligand 5,6‐dimethylbenzimidazole (DMBI) or its natural precursors (riboflavin and nicotinamide) on active B12 production was evaluated. Active B12 production was confirmed by UHPLC–UV–MS analysis. A B12 content of 12–37 μg·kg^−1^ was produced in BM; this content increased 10‐fold with cobalt and reached 940–1,480 μg·kg^−1^ with both cobalt and DMBI. With riboflavin and nicotinamide, B12 production in cobalt‐supplemented BM increased to 712 μg·kg^−1^. Approximately, 10 μg·kg^−1^ was achieved in BF and AM and was increased to 80 μg·kg^−1^ in BF and 260 μg·kg^−1^ in AM with cobalt and DMBI. The UHPLC and microbiological assay (MBA) results agreed when both cobalt and DMBI or riboflavin and nicotinamide were supplemented. However, MBA gave ca. 20%–40% higher results in BM and AM supplemented with cobalt, indicating the presence of human inactive analogues, such as pseudovitamin B12. This study demonstrates that cereal products can be naturally fortified with active B12 to a nutritionally relevant level by fermenting with *P. freudenreichii*.

## 1 INTRODUCTION

Vitamin B12 (hereafter called B12) is naturally present only in foods of animal origin and through processing in certain fermented plant foods or fortified products (Truswell, 2007; Watanabe, Yabuta, Tanioka, & Bito, 2013). According to a review by the WHO (2008), B12 and folate deficiencies may be public health problems worldwide. B12 deficiency is particularly widespread in developing countries due to an inadequate consumption of animal‐based foods (Allen, 2009; Marsh, Zeuschner, & Saunders, 2012). In wealthier countries, vegetarians, vegans, and the elderly are at risk of deficiency (Allen, 2010; Pawlak, Lester, & Babatunde, 2014). Moreover, the current interest in substituting animal proteins with plant proteins could reduce dietary B12 intake in the future (Elmadfa & Singer, 2009; Marsh et al., 2012). Therefore, a sustainable solution to address B12 deficiency could be the fortification of plant‐based products, preferably by natural means, such as fermentation fortification with B12‐producing food‐grade microorganisms.

Some efforts have been made to enrich plant‐derived foods, such as tempeh (Keuth & Bisping, 1994; Liem, Steinkraus, & Cronk, 1977; Mo et al., 2013) and fermented vegetables (Babuchowski, Laniewska‐Moroz, & Warminska‐Radyko, 1999) with B12. The reported B12 content in these products varies considerably (1.6–80 μg·kg^−1^ fresh weight) depending on the microorganisms involved and the fermentation conditions applied (Mo et al., 2013; Watanabe et al., 2013). In tempeh fermentation, the microorganisms responsible for B12 production are the contaminants *Citrobacter freundii* and *Klebsiella pneumoniae* (Keuth & Bisping, 1994; Watanabe et al., 2013). Recently, Gu, Zhang, Song, Li, & Zhu (2015) claimed a B12 content up to 180 μg·L^−1^ in soy yoghurt fermented with *Lactobacillus reuteri* strain ZJ03. Earlier, feeding soymilk fermented with *L. reuteri* 1098 was shown to correct the symptoms of B12 deficiency in pregnant mice and their offspring (Molina, Médici, de Valdez, & Taranto, 2012). The B12 in these studies was analyzed with the microbiological assay (MBA), which lacks the specificity to distinguish the active form of B12 [with 5,6‐dimethylbenzimidazole (DMBI) as the lower ligand) from its human inactive analogues (with non‐DMBI lower ligands) (Watanabe et al., 2013). In particular, the characterization of the type of the B12 compound in fermented products is important because several microorganisms produce B12 analogues that are biologically inactive for humans (Watanabe et al., 2013). For example, *L. reuteri* exclusively synthesizes pseudovitamin B12 with adenine as the lower ligand (Crofts, Seth, Hazra, & Taga, 2013; Santos et al., 2007), and this B12 analogue is known to be inactive in humans (Stupperich & Nexø, 1991; Watanabe et al., 2013). Liquid chromatographic methods are better suited for the measurement of active B12 in food materials, particularly for samples containing the B12 analogues in which the MBA analysis is unreliable (Kumar, Chouhan, & Thakur, 2010). In our earlier work (Chamlagain, Edelmann, Kariluoto, Ollilainen, & Piironen, 2015), we reported a sensitive and selective method suitable for the determination of active B12 in fermented products, using ultra‐high performance liquid chromatography (UHPLC) after purifying sample extracts on B12‐specific immunoaffinity columns.

*Propionibacterium freudenreichii* is the only generally recognized as safe (GRAS) B12‐synthesizing bacterium (Thierry et al., 2011). The use of *P. freudenreichii* in cereal‐based products (e.g., bread) is currently limited to improving the product shelf life (Suomalainen & Mäyrä‐Makinen, 1999; Tinzl‐Malang, Rast, Grattepanche, Sych, & Lacroix, 2015). However, our recent studies indicated the organisms' abilities to produce active B12 in cereal‐based matrices (Chamlagain et al., 2015; Edelmann, Chamlagain, Santin, Kariluoto, & Piironen, 2016). In *P. freudenreichii,* the availability of cobalt and the B12 lower ligand DMBI was shown to be critical to improving active B12 production (Hugenschmidt, Schwenninger, & Lacroix, 2011). DMBI use is not allowed in food production. However, DMBI supplementation is a common practice in pharmaceutical B12 production (Martens, Barg, Warren, & Jahn, 2002). In a recent study, we showed that enhanced B12 production by *P. freudenreichii* in a whey‐based medium was possible by substituting DMBI with riboflavin (RF) and nicotinamide (NAM) co‐supplementation (Chamlagain et al., 2016). For natural fortification, avoiding DMBI supplementation for improved B12 production in plant‐based foods by substituting with natural precursors RF and NAM is of significant interest. Additionally, the low cobalt content of cereal materials (10–60 μg·kg^−1^) (Ekholm et al., 2007; Varo, Nuurtamo, Saari, & Koivistoinen, 1980) may still be a bottleneck for enhancing B12 production in cereal‐based matrices.

In this study, we investigated the in situ production of active B12 in three different aqueous cereal matrices by fermentation with food‐grade *P. freudenreichii* strains and supplementation of cobalt, and DMBI or its precursors RF and NAM. We used UHPLC combined with high‐resolution mass spectrometry (MS/MS) for the reliable identification and quantification of active B12, using MBA as a reference method.

## 2 MATERIALS AND METHODS

### 2.1 Chemicals and solvents

The following materials were obtained from Sigma‐Aldrich (Steinheim, Germany): acetonitrile (HPLC grade), Tween 80, sodium cyanide, trifluoroacetic acid (TFA), RF, NAM and cobalt(II) chloride (Co). Sodium hydroxide, acetic acid, and DMBI were purchased from Merck (Darmstadt, Germany). Cyanocobalamin, which was used as a standard in both UHPLC and MBA, was obtained from Supelco (Bellefonte, PA, USA). The water (later called MilliQ‐water) used in the fermentation experiments and analyses was obtained from the MilliQ Plus system (0.22 μm, ≥18.2 MΩ·cm; Millipore Corporation, Bedford, MA, USA).

### 2.2 Preparation of the cereal matrices

With an aim to maximize the flour components but to maintain workable viscosity, the proportion of flour in the matrices (w/v) was 33% in the barley malt matrix (BM), 6% in the barley flour matrix (BF), and 15% in the wheat aleurone matrix (AM). Malted barley flour (milled malted barley grains; Laihian Mallas Ltd., Finland), commercial barley flour (Myllyn Paras Ltd., Finland), or high‐purity wheat aleurone (Bühler AG, Switzerland) was mixed with boiling MilliQ‐water and cooked for 2 min. The cooked matrices were transferred into Erlenmeyer flasks and then autoclaved (121°C; 15 min). Prior to the experiment, the matrices were aseptically weighed (20 g) in 50 ml Falcon tubes.

### 2.3 Preparation of *P. freudenreichii* cultures

Three dairy‐originated *P. freudenreichii* strains (designated 256, 263, and 266) were selected based on their differing capabilities to produce B12 (Chamlagain et al., 2016). First, the cryopreserved cultures (−80°C) were transferred to propionic agar medium (Chamlagain et al., 2016). Then, three individual colonies of each strain were subcultured separately three times in whey‐based medium (Chamlagain et al., 2016) under anaerobic conditions (Anaerocoult; Merck, Germany) at 30°C for 3–4 days.

### 2.4 Addition of supplements and fermentation of the matrices

The matrices were studied for B12 production with and without the addition of Co, Co in combination with DMBI or Co co‐supplemented with RF and NAM (Table 1). The supplements Co (5 mg·kg^−1^ medium), RF (15 mg·kg^−1^ medium) and NAM (3 g·kg^−1^ medium) were added on day 0, whereas DMBI (15 mg·kg^−1^ medium) was provided after 144 hr of incubation (Chamlagain et al., 2016). Each matrix with or without supplements was studied with three biological replicate cultures of each strain. The replicate cultures were inoculated (1%) to achieve an initial cell count of 8.0 log CFU·g^−1^ of matrix. The matrices were incubated at 30°C for 168 hr [72 hr anaerobically without shaking and then under the microaerobic condition with shaking (150 rpm)]. AM was studied only with strains 256 and 266.

**Table 1:** The change in pH values and the final cell counts (Log CFU·g^‐1^ matrix) of the cereal matrices fermented with three *P. freudenreichii* strains (256, 263, or 266) without or with supplements (Co, RF and NAM or DMBI) during 168 hr of incubation at 30°C

Strain	Malt matrix + supplement	pH		Cell counts (log CFU·g^−1^) 168 hr	Flour matrix + supplement	pH		Cell counts (log CFU·g^−1^) 168 hr	Aleurone matrix + supplement	pH		Cell counts (log CFU·g^−1^) 168 hr
		0 hr	168 hr			0 hr	168 hr			0 hr	168 hr	
256	BM	5.62	4.61	9.71 ± 0.05	BF	5.70	5.70	8.85 ± 0.05	AM	6.61	6.56	9.27 ± 0.01
	BM Co		4.48	9.80 ± 0.10	BF Co		5.57	8.90 ± 0.22	AM Co		6.51	9.23 ± 0.03
	BM Co + RF + NAM		4.51	9.76 ± 0.11	BF Co + RF + NAM^a^				AM Co + RF + NAM		6.51	9.16 ± 0.03
	BM DMBI		4.62	9.73 ± 0.02	BF DMBI		5.75	8.78 ± 0.34	AM DMBI^a^			
	BM DMBI + Co		4.62	9.73 ± 0.10	BF DMBI + Co		5.75	8.75 ± 0.16	AM DMBI + Co		6.57	9.22 ± 0.10
263	BM	5.61	4.55	9.76 ± 0.05	BF	5.68	5.67	8.97 ± 0.13	AM^a^			
	BM DMBI		4.53	9.70 ± 0.04	BF DMBI		5.68	9.04 ± 0.15	AM DMBI^a^			
	BM DMBI + Co		4.54	9.84 ± 0.02	BF DMBI + Co		5.68	8.94 ± 0.12	AM DMBI + Co^a^			
266	BM	5.63	4.49	9.74 ± 0.15	BF	5.72	5.55	8.86 ± 0.10	AM	6.61	6.45	9.32 ± 0.14
	BM Co		4.41	9.71 ± 0.04	BF Co		5.74	8.95 ± 0.11	AM Co		6.44	9.29 ± 0.12
	BM Co + RF + NAM		4.60	9.76 ± 0.15	BF Co + RF + NAM^a^				AM Co + RF + NAM		6.45	9.29 ± 0.10
	BM DMBI		4.49	9.84 ± 0.05	BF DMBI		5.57	8.90 ± 0.05	AM DMBI^a^			
	BM DMBI + Co		4.53	9.76 ± 0.05	BF DMBI + Co^a^				AM DMBI + Co		6.52	9.36 ± 0.05

Co, cobalt chloride; RF, riboflavin; NAM, nicotinamide; DMBI, 5,6‐dimethylbenzimidazole; BM, barley malt matrix; BF, barley flour matrix; AM, wheat aleurone matrix.

Cell counts data are mean ± SD of three biological replicate fermentations.

a Not studied.

### 2.5 Monitoring of fermentation

The change in the number of bacterial cells during fermentation was followed by viable cell counting (CFU·g^−1^) at the beginning and after 168 hr of incubation. For enumeration of the cell numbers, the samples were serially diluted (with phosphate buffered saline, 0.9% w/v; Oxoid, Hampshire, UK) and then inoculated on propionic agar medium and incubated anaerobically for 3–4 days at 30°C. The pH of the matrices before and after fermentation was measured with a pH metre (MeterLab; Radiometer Analytical, Lyon, France).

### 2.6 Vitamin B12 analysis

The B12 content of the fermented cereal matrices was determined after extraction as cyanocobalamin by the UHPLC–UV and MBA methods as previously reported (Chamlagain et al., 2015). The identification was confirmed by analyzing the extracts with a high‐resolution mass spectrometer. The B12 amounts (calculated as μg·kg^−1^ matrix) are averages from three biological replicate fermentations, each of which was analyzed as a single replicate.

#### 2.6.1 Extraction

Fermented matrix (1–3 g) was vortexed with 10 ml of extraction buffer (8.3 mmol/L sodium hydroxide and 20.7 mmol/L acetic acid; pH 4.5) and 100 μl of 1% sodium cyanide. Following incubation (37°C; 40 min) with α‐amylase (50 mg), the sample was boiled for 30 min. After cooling on ice, the supernatant was separated by centrifugation (6,900*g*, 10 min). The pellet was vortexed with 5 ml of the extraction buffer (pH 6.2) and recentrifuged. The combined supernatants were adjusted to pH 6.2, paper‐filtered and diluted to 25 ml with the pH 6.2 buffer. The extracts were analyzed for B12 with MBA after appropriate dilution and with the UHPLC method after purification on the immunoaffinity columns.

#### 2.6.2 Immunoaffinity purification

Sample extract (5–15 ml) was eluted through an immunoaffinity column (Easy‐Extract; R‐Biopharma, Glasgow, UK). Then, the column was washed with 10 ml of MilliQ‐water, and the analyte was recovered with methanol (3 + 0.5 ml). The eluate was evaporated under a nitrogen stream at 60°C, and the residue was reconstituted in 300 μl of MilliQ‐water. Finally, the extracts were syringe‐filtered (0.2 μm; Pall, USA).

#### 2.6.3 UHPLC and LC–MS analyses

The UHPLC analysis was performed on a Waters Acquity UPLC system (Milford, MA, USA) equipped with a photodiode array detector (PDA; 210–600 nm), using an Acquity HSS T3 C18 column (Waters; 2.1 × 100 mm; 1.8 μm). The absorption spectra were recorded, and a chromatogram at 361 nm was acquired. A gradient flow of MilliQ‐water and acetonitrile (both modified with 0.025% TFA) was run for 10 min (Chamlagain et al., 2015). The B12 concentration of the samples was calculated, using an external calibration curve obtained by injecting cyanocobalamin standards (0.04–0.4 mg·L^−1^). Both the samples and standards were injected twice (10 μl).

The purified extracts were further analyzed to confirm the identity of cobamide with a high‐resolution quadrupole time‐of‐flight mass spectrometer with electrospray ionization (Q‐TOF, Synapt G2‐Si; Waters, MA, USA) interfaced to the Waters UPLC. For the MS analysis, TFA in the mobile phase was replaced with formic acid (0.1%). The ionization was operated in the positive ion mode with the *m*/*z* range set for 50–1,500. Protonated molecular parent ions were detected, isolated, and fragmented (MS/MS), using argon as the collision gas. The following instrument settings were applied (Deptula et al., 2015): capillary voltage 0.5 kV, sampling cone voltage 40 V, source offset 80 V, source temperature 150°C, desolvation temperature 600°C, desolvation gas flow 1,000 L·h^−1^, nebulizer gas flow 6.5 bar, cone gas flow 50 L·h^−1^, trap collision energy 4 eV, ramp trap collision energy 15–90 eV, trap gas flow 2 ml·min^−1^ and scan time of 0.2 s.

#### 2.6.4 Microbiological assay

The MBA was performed on a microtiter plate, using *Lactobacillus delbrueckii* ATCC 7830 as an assay organism according to the previously reported method (Chamlagain et al., 2015). A certified reference material (lyophilized pig liver; B12 content: 1,120 ± 90 μg·kg^−1^ dm; Institute for Reference Materials and Measurements, Geel, Belgium) was included in each assay (the analytical average was 1,130 ± 65 μg·kg^−1^dm).

### 2.7 Determination of sugars and acids

The fermented and control matrices were mixed with MilliQ‐water (1:10), using an Ultra Turrax homogenizer (IKA, Germany) at 9,500 rpm for 30 s. The supernatants were separated by centrifugation (6,900*g*, 10 min) and syringe‐filtered (0.45 μm) into HPLC vials. The acids and sugars were separated, using an Aminex HPX‐87H column (7.8 × 300 mm, 9 μm; Bio‐Rad, USA) and detected with the UV (210 nm; acids) and refractive index (sugars) detectors on a Waters HPLC system (Hugenschmidt, Schwenninger, Gnehm, & Lacroix, 2010). An isocratic flow of 10 mmol/L sulfuric acid (0.6 ml·min^−1^) was set through the column maintained at 40°C. Both the samples and standards (40 μl) were injected twice. For quantitation, calibration curves obtained by injecting standards containing glucose, fructose, maltose, propionic acid, and acetic acid (0.04–1.0 g·L^−1^) were employed.

### 2.8 Statistical analysis

One‐way analysis of variance with Tukey's post hoc test (using SPSS version 23, IBM Inc., Chicago, IL, USA) was performed, with a *p* value <.05 considered statistically significant. The effects of supplements on the cell counts (CFU·g^−1^) and B12 yield (μg·kg^−1^) were compared. The B12 results obtained with UHPLC and MBA were also statistically evaluated.

## 3 RESULTS AND DISCUSSION

### 3.1 Fermentation characteristics

BM and BF were fermented with all three *P. freudenreichii* strains, whereas AM was fermented only with strains 256 and 266 (Table 1). During BM fermentation by each of the three strains (256, 263, or 266), the initial cell counts (8.0 log CFU·g^−1^) increased to approximately 10.0 log CFU·g^−1^ after 168 hr of incubation (Table 1). This 100‐fold rise in viable cell numbers was accompanied by a decrease in the pH of the matrix from an initial value of 5.6 to 4.6–4.4. The cell counts in BF increased by ten‐fold (9.0 log CFU·g^−1^) and were slightly lower than the cell counts in AM (9.3 log CFU·g^−1^). Unlike BM, the pH of BF and AM remained unchanged after 168 hr of incubation (Table 1), which was in agreement with the limited growth in the nonmalt matrices. The strains grew to similar cell densities in the respective matrices after 168 hr of fermentation (*p* > .05). Additionally, supplementation of the matrices with Co, DMBI, or RF and NAM did not affect the final cell counts (*p* > .05) or the pH of the matrices (Table 1).

In the fermented BM, the fermentation metabolites propionic acid and acetic acid were 4.2–5.4 and 3.2–4.0 g·kg^−1^ medium, respectively (Table 2). Total acid production was not affected by the addition of the B12 precursors. In line with the more limited growth in BF and AM compared with BM, the level of acids produced during BF or AM fermentation was <0.2 g·kg^−1^ medium. Glucose was the main fermentable sugar for the *P. freudenreichii* strains in BM (8 g·kg^−1^ medium) and was found completely depleted after 168 hr of incubation. The maltose content of the matrices was not affected by the fermentation (data not shown), which was consistent with the inability of *P. freudenreichii* strains to metabolize maltose (Loux et al., 2015). The fermentable sugars (glucose and fructose) in BF and AM were quite low (<0.2 g·kg^−1^ medium). The better growth of *P. freudenreichii* strains and their higher metabolite production in BM was likely due to the better availability of the carbon sources and other nutrients as BM contained 33% flour components (w/v) compared to 6% (w/v) in BF and 15% (w/v) in AM.

**Table 2:** Propionic acid (PA) and acetic acid (AA) contents (g·kg^−1^ matrix) in barley malt matrix (BM) fermented for 168 hr by *P  freudenreichii* strains 256, 263, and 266 without or with Co, RF, and NAM or DMBI supplementation

Strain	Matrix + supplement	PA g·kg^−1^	AA g·kg^−1^
256	BM	4.5 ± 0.17	3.6 ± 0.11
	BM Co	4.7 ± 0.24	4.0 ± 0.24
	BM Co + RF + NAM	4.9 ± 0.40	3.5 ± 0.29
	BM DMBI	4.6 ± 0.28	3.6 ± 0.34
	BM DMBI + Co	4.4 ± 0.10	3.6 ± 0.03
263	BM	4.8 ± 0.09	3.7 ± 0.08
	BM DMBI	5.1 ± 0.18	3.9 ± 0.20
	BM DMBI + Co	4.9 ± 0.06	4.0 ± 0.12
266	BM	4.2 ± 0.32	3.2 ± 0.21
	BM Co	4.7 ± 0.40	4.0 ± 0.30
	BM Co + RF + NAM	5.4 ± 0.17	3.7 ± 0.20
	BM DMBI	4.6 ± 0.10	3.8 ± 0.13
	BM DMBI + Co	4.3 ± 0.15	3.4 ± 0.01

Co, cobalt chloride; RF, riboflavin; NAM, nicotinamide; DMBI, 5,6‐dimethylbenzimidazole.

### 3.2 UHPLC and LC–MS identification of vitamin B12

**Figure 1:**
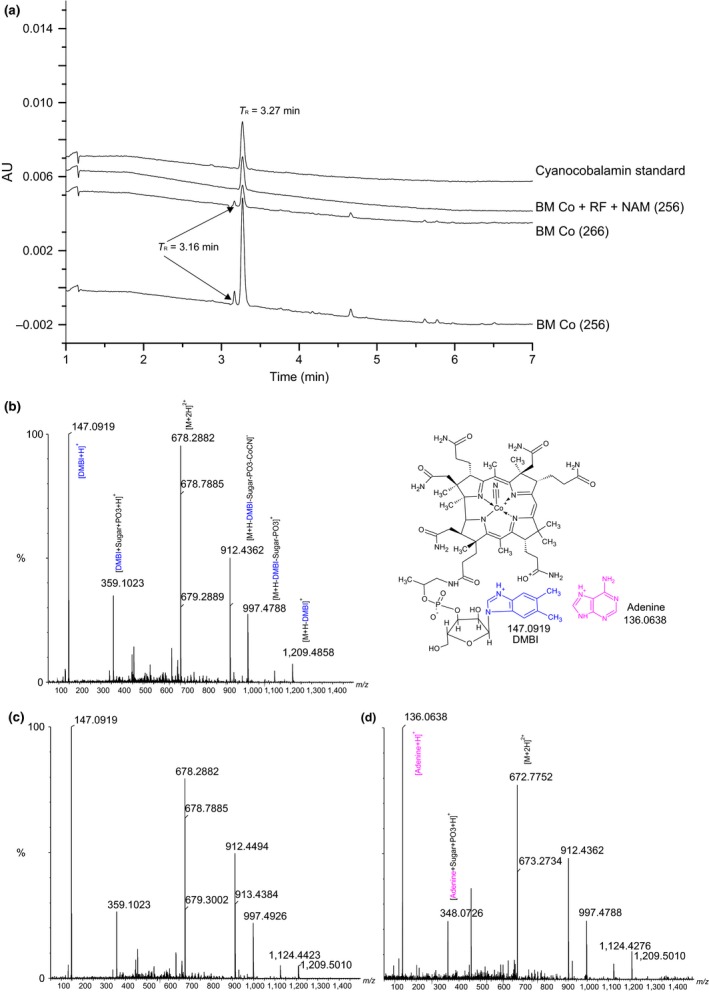
Example UHPLC–UV chromatograms showing a cyanocobalamin peak (3.27 min) from a cyanocobalamin standard and immunoaffinity purified extracts of the barley malt matrix (BM) supplemented with Co or Co together with RF and NAM and fermented with *Propionibacterium freudenreichii* 256 or 266 (a). Example Q‐TOF–MS/MS spectra of the cyanocobalamin peak (3.27 min) from the standard (b) and fermented matrix supplemented with Co (c) and the peak at 3.16 min (Figure A) identified as pseudovitamin B12 in the Co‐supplemented fermented BM (d). Co, cobalt chloride; RF, riboflavin; NAM, nicotinamide; M, cyanocobalamin

In Figure 1, we show the UHPLC chromatograms and Q‐TOF mass spectra of the cyanocobalamin standard and the immunoaffinity‐purified extracts of representative matrices fermented with strains 256 and 266. Extracts of the fermented BM with Co and with Co together with RF and NAM resulted in a peak at 3.27 min (UV 361 nm), which eluted at exactly the retention time of cyanocobalamin (Figure 1a). The PDA spectra (210–600 nm) were identical to those of cyanocobalamin (data not shown). In the MS analysis, the cobamide peak produced ions with an *m*/*z* of 678.2882; the *m*/*z* value perfectly matched that of positively doubly charged cyanocobalamin ([M + 2H]^2+^). Parent ions with the same *m*/*z* were detected when the cyanocobalamin standard was analyzed. On performing MS/MS, fragment ions with an *m/z* of 1209.4858, 1124.4423, 997.4788, 912.4494, 678.2882, 359.1023, and 147.0919 (Figure 1c) were obtained; these values were identical to those of the cyanocobalamin fragmentation products (Figure 1b). Similarly, cyanocobalamin was detected in the fermented BM, BF and AM extracts with and without added supplements. Overall, the UHPLC–PDA and LC−MS analysis results confirmed that the cereal matrices fermented with *P. freudenreichii* strains 256, 263, and 266 contained active B12 with DMBI as the lower ligand (*m*/*z* 147.0919 [DMBI + H]^+^). Moreover, a minor pseudovitamin B12 peak (with adenine as the lower ligand) was also observed in the fermented BM extracts supplemented with Co (Figure 1d).

### 3.3 B12 production in the cereal matrices

**Figure 2:**
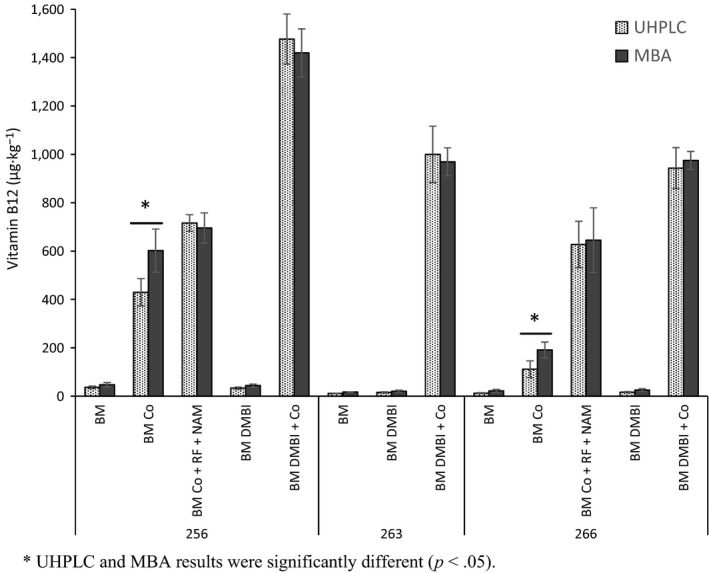
Vitamin B12 production by the *P. freudenreichii* strains (256, 263 and 266) in the malted barley matrix (BM) without or supplemented with cobalt chloride (Co), riboflavin (RF), and nicotinamide (NAM), or 5,6‐dimethylbenzimidazole (DMBI). The results were obtained with UHPLC–UV and MBA and are given as averages of three biological replicate fermentations (error bars represent standard deviations)

The B12 production levels in the matrices with and without supplementation by *P. freudenreichii* strains 256, 263, and 266 obtained with the UHPLC method and MBA are shown in Figure 1 (BM) and Figure 2 (BF and AM). We evaluate and discuss B12 production based on the UHPLC results and later compare these results with those obtained from MBA.

#### 3.3.1 B12 production without supplementation

**Figure 3:**
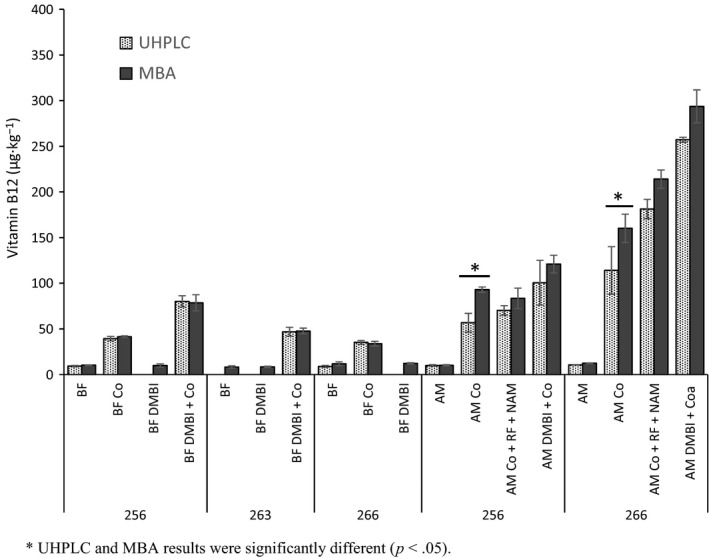
Vitamin B12 production by *P. freudenreichii* strains 256, 263, and 266 in barley flour matrix (BF) and aleurone matrix (AM) without or with cobalt chloride (Co), riboflavin (RF), and nicotinamide (NAM) or 5,6‐dimethylbenzimidazole (DMBI) supplementation. The results were obtained by UHPLC and MBA and are given as averages of three biological replicate fermentations, with error bars representing the standard deviations

Among the three strains, the highest active B12 production in BM without any supplements (37 μg·kg^−1^) was obtained with strain 256. The production in this strain was ca. threefold higher than the yield obtained with strain 263 or 266 (12–13 μg·kg^−1^) (Figure 2). B12 production in BF and AM was 9–10 μg·kg^−1^ in the studied strains (Figure 3). The flour level differed in the matrices (33% w/v in BM vs. 6% in BF vs. 15% in AM), and the flours varied in the amounts of fermentable sugars and B12‐precursors. Our RF analysis (Chamlagain et al., 2016) of the raw materials showed that wheat aleurone contained a higher RF level (3.1 mg·kg^−1^) than the malted barley flour (2.3 mg·kg^−1^), whereas the amount of RF in the barley flour was considerably low (0.5 mg·kg^−1^). Thus, the estimated RF concentration in the matrices would differ significantly (BM, 0.6 mg·kg^−1^; BF, 0.03 mg·kg^−1^; and AM, 0.4 mg·kg^−1^). The matrices also differed in the cobalt (Varo et al., 1980) and niacin contents (Buri, Von Reding, & Gavin, 2004; Hucker, Wakeling, & Vriesekoop, 2012). Therefore, the strain and the matrix compositions (particularly the cobalt, RF, and fermentable sugar levels) most likely affected B12 production in the basic matrices (Figures 2 and 3). Nevertheless, the level of produced active B12 in these matrices without any added supplements (9–37 μg·kg^−1^) was at a nutritionally significant level. The recommended dietary allowance (RDA) for B12 for adults is 2.4 μg/day (Institute of Medicine, 1998) or 2.0 μg/day (Nordic Nutrition Recommendations 2012, 2013); thus, the amount of B12 produced in the basic matrices when used in a food product could easily fulfill the RDA.

#### 3.3.2 Effect of supplementation on B12 production

B12 production in the matrices with the supplements was dependent on the matrix and the *P. freudenreichii* strain. A considerably higher amount of active B12 was produced in nutrient‐rich BM compared to AM and BF by the supplementation of Co or its combination with DMBI or RF and NAM (Figures 2 and 3).

As shown in Figure 2, Co supplementation increased B12 production in BM by strain 256 from 37 μg·kg^−1^ to 430 μg·kg^−1^, whereas the yield of strain 266 increased from 13 μg·kg^−1^ to 130 μg·kg^−1^. The Co addition in BF improved the B12 yield ca. threefold to 39 μg·kg^−1^ (with 256) and 35 μg·kg^−1^ (with 266) from the 10 μg·kg^−1^ in the basic BF (Figure 3). In AM, the B12 production was increased to 57 μg·kg^−1^ with 256 and 114 μg·kg^−1^ with 266 by Co supplementation. These results showed that an improved fermentation fortification of cereal matrices with active B12 is possible by simply increasing the Co level in the matrices.

However, DMBI supplementation alone did not increase B12 production by any of the strains in the BM and BF (Figures 2 and 3; AM was not studied with DMBI). In contrast, when DMBI was added to Co‐supplemented BM, the B12 yield by all three *P. freudenreichii* strains increased several fold (Figure 2). The production by strain 256 reached as high as 1,480 μg·kg^−1^ (40‐fold increase), whereas the yield improved even more for strains 263 (943 μg·kg^−1^; 86‐fold) and 266 (1,000 μg·kg^−1^; 75‐fold). In BF, the B12 production was increased moderately by both DMBI and Co supplementation (Figure 3). B12 production of 80 μg·kg^−1^ was achieved with strain 256 and 47 μg·kg^−1^ with strain 263. DMBI and Co supplementation in AM was more effective for strain 266 than for *P. freudenreichii* strain 256, with an ~24‐fold higher yield obtained with DMBI and Co with strain 266 (257 μg·kg^−1^) as opposed to only 10‐fold with strain 256 (101 μg·kg^−1^) from their respective B12 production levels in the basic matrices (Figure 3).

Earlier studies showed that B12 production by *P. freudenreichii* in whey‐based media was affected by the Co and DMBI availability in a strain‐dependent manner (Chamlagain et al., 2016; Hugenschmidt et al., 2011). Cereal‐based matrices have not been studied previously for fermentation fortification of B12. However, the fermented soy product tempeh has been investigated for B12 enrichment with *C. freundii* or *K. pneumoniae* (Denter & Bisping, 1994; Keuth & Bisping, 1993, 1994; Wiesel, Rehm, & Bisping, 1997). By supplementation with Co or DMBI, the B12 content of tempeh was improved ~twofold to ca. 300 μg·kg^−1^ dry weight. However, B12 production with Co and DMBI co‐supplementation was not performed in the study of Keuth and Bisping (Keuth & Bisping, 1994).

Because the use of DMBI in food production is not possible, we considered the use of its natural alternatives (RF and NAM) to improve active B12 production in the cereal matrices. B12 production in the Co‐enriched matrices was further increased by supplementing with RF and NAM (Figures 2 and 3), the level of B12 produced was, however, lower than that achieved with DMBI supplementation. This effect was studied in BM and AM with *P. freudenreichii* strains 256 and 266. When RF and NAM were supplemented with Co, the B12 yield in BM improved by both of these strains (Figure 2), reaching up to 716 μg·kg^−1^ with strain 256 and 627 μg·kg^−1^ with strain 266. RF and NAM supplementation was more effective for strain 266 considering its lower yield with Co alone (114 μg·kg^−1^). In AM, the yield improved slightly with strain 256 (1.2‐fold) and 1.6‐fold with strain 266 following RF and NAM supplementation (Figure 3). These results are in agreement with our previous study in which we showed that RF and NAM could be food‐grade substitutes for DMBI to enhance B12 production by *P. freudenreichii* strains (Chamlagain et al., 2016).

Overall, the results obtained in this study suggest that RF and NAM can be used to enhance the *in situ* production of active B12 in cereal matrices. Earlier, we showed that B12 production by *P. freudenreichii* strains correlated with the depletion of RF from a whey‐based medium, whereas co‐supplementation of RF and NAM‐enhanced B12 production in a strain‐dependent manner (Chamlagain et al., 2016). However, RF from different matrices may differ due to its existence as free or bound to proteins (Ball, 2006; Ndaw, Bergaentzlé, Aoudé‐Werner, & Hasselmann, 2000). Therefore, the accessibility of RF from cereal matrices for *de novo* DMBI biosynthesis by the *P. freudenreichii* strains could have affected B12 production in the matrices without its supplementation. In *P. freudenreichii*, the DMBI ligand of B12 is derived from RF, and one of the biosynthetic steps requires oxygen (Hörig & Renz, 1977; Hörig, Renz, & Heckmann, 1978), a microaerophilic condition was, therefore, maintained after 72 hr of the 7‐day fermentation. NAM was shown to stimulate DMBI synthesis from RF (Hörig & Renz, 1980), leading to enhanced B12 production in *P. freudenreichii* (Chamlagain et al., 2016).

### 3.4 Comparison of the UHPLC results with MBA

The UHPLC analysis results of the B12 contents of the fermented basic matrices and with DMBI supplementation were similar to those obtained with MBA (*p* > .05; Figures 2 and 3). However, the MBA results for BM and AM supplemented with Co were up to 40% higher than the results obtained with the UHPLC method (*p* < .05). In BF fermented with Co, where the B12 level was <85 μg·kg^−1^, both methods produced comparable results (Figure 3). The addition of both Co and DMBI increased B12 production considerably, and the UHPLC and MBA results matched (*p* > .05; Figures 2 and 3). Likewise, when DMBI was substituted with RF and NAM in the Co‐supplemented matrices, the B12 results obtained by MBA and UHPLC agreed (*p* > .05).

These results clearly show that B12 production by the *P. freudenreichii* strains is enhanced and directed toward the biosynthesis of active B12 when the necessary precursors for B12 biosynthesis are abundant. Because Co is inserted early in the anaerobic B12 synthesis pathway in *P. freudenreichii* (Friedman & Cagen, 1970; Moore & Warren, 2012), the availability of Co directly affected the production of active B12 in the matrices. Furthermore, without sufficient *de novo* synthesis of DMBI or its exogenous supplementation, B12 synthesis could remain incomplete or lead to the synthesis of cobamides with a lower ligand other than DMBI (Deptula et al., 2015; Vorobjeva, 1999). In this study, one such cobamide was detected, albeit in a low amount, in Co‐supplemented BM fermented with strains 256 and 266 (Figure 1a; retention time = 3.16 min). By MS/MS we confirmed this cobamide to be pseudovitamin B12 with adenine (*m*/*z* 136.0638 [M + H]^+^) as its lower ligand (Figure 1d). Alternate cobamides were shown to support the growth of the assay organism *L. delbrueckii* used in the MBA for B12 analysis, thereby overestimating the active B12 level in these samples (Watanabe, 2007). The higher B12 contents measured with MBA compared to the actual amounts of active B12 detected by UHPLC indicate the likely presence of inactive corrinoids in the fermented matrices. This study and our previous study (Chamlagain et al., 2015) suggest that the B12 contents of fermented food materials obtained with MBA need confirmation with more accurate methods, such as UHPLC.

## 4 CONCLUSION

A nutritionally significant level of active B12 was produced in cereal matrices (9–37 μg·kg^−1^; RDA: 2–2.4 μg/day) without supplementation by *P. freudenreichii* strains. Furthermore, the B12 production in these matrices was increased several fold (more effectively in BM) by supplementation with Co and DMBI or its food‐grade precursors RF and NAM. In BM supplemented with Co, B12 production of 943–1,480 μg·kg^−1^ with DMBI and up to 627–712 μg·kg^−1^ with RF and NAM was achieved. In AM with Co, the B12 yield with RF and NAM was up to 181 μg·kg^−1^. The results indicate that RF and NAM are food‐grade alternatives to DMBI supplementation to enhance active B12 production in food matrices. The B12 results obtained with UHPLC and MBA were identical with the exception of the results obtained for fermented BM and AM supplemented with Co. The 20%–40% higher B12 contents obtained with MBA in the Co‐supplemented matrices indicate the presence of inactive cobamides (e.g., pseudovitamin B12), which was detected in BM. By further modifying and optimizing the matrix composition and fermentation conditions, improved B12 production in cereal matrices could be possible. Overall, this study shows that cereal‐based matrices are suitable for the natural B12 fortification of plant‐based foodstuffs or food ingredients by fermentation with the food‐grade bacterium *P. freudenreichii*.

## CONFLICT OF INTEREST

None declared.
